# Alabama

**Published:** 1896-05

**Authors:** 


*Alabama.* Montgomery has passed a city ordinance (to take effect October 1, 1896), establishing a public slaughter-house, at which all animals must be slaughtered. All animals must be inspected on foot and at the slaughter by an inspector, who must be a veterinarian. The ordinance also provides for inspection of all dairy herds which supply milk to the city.

